# A New Species of Eagle Ray *Aetobatus narutobiei* from the Northwest Pacific: An Example of the Critical Role Taxonomy Plays in Fisheries and Ecological Sciences

**DOI:** 10.1371/journal.pone.0083785

**Published:** 2013-12-31

**Authors:** William T. White, Keisuke Furumitsu, Atsuko Yamaguchi

**Affiliations:** 1 CSIRO Marine & Atmospheric Research, Wealth from Oceans Flagship, Hobart, Tasmania, Australia; 2 Faculty of Fisheries, Nagasaki University, Nagasaki, Japan; University of Bologna, Italy

## Abstract

Recent taxonomic and molecular work on the eagle rays (Family Myliobatidae) revealed a cryptic species in the northwest Pacific. This species is formally described as *Aetobatus narutobiei* sp. nov. and compared to its congeners. *Aetobatus narutobiei* is found in eastern Vietnam, Hong Kong, China, Korea and southern Japan. It was previously considered to be conspecific with *Aetobatus flagellum*, but these species differ in size, structure of the NADH2 and CO1 genes, some morphological and meristic characters and colouration. *Aetobatus narutobiei* is particularly abundant in Ariake Bay in southern Japan where it is considered a pest species that predates heavily on farmed bivalve stocks and is culled annually as part of a ‘predator control’ program. The discovery of *A. narutobiei* highlights the paucity of detailed taxonomic research on this group of rays. This discovery impacts on current conservation assessments of *A. flagellum* and these need to be revised based on the findings of this study.

## Introduction

The extinction risks facing chondrichthyan fishes (sharks, skates, rays and chimaeras) is far higher than for most other vertebrate groups and up to one-quarter are threatened due to overfishing [1]. A large proportion of the species included in the IUCN Red List of Threatened Species are listed as Data Deficient due to lack of scientific knowledge, particularly taxonomic, for those species. Good taxonomic research on chondrichthyan fishes is a fundamental requirement and forms the foundation for all other life sciences [2]. Despite this, published studies on chondrichthyans focus on ecological aspects but for most species, even basic taxonomic investigations are rarely undertaken. A good example of the critical role taxonomy plays in the life sciences is that of the common skate *Dipturus batis* in the Eastern Atlantic. *Dipturus batis* is a large skate species which has been described as the first case of a fish species brought to the brink of extinction by overfishing [3]. However, a taxonomic revision of this species by [4] concluded that two species were confused under the one name. Thus, previous ecological studies on this species include data from two sympatric species which cannot be disentangled. The present study investigates the identity of a northwest Pacific eagle ray species, previously considered conspecific with *Aetobatus flagellum*, to highlight the crucial fundamental role taxonomy plays in the life sciences.

The longheaded eagle ray, *A. flagellum* Bloch & Schneider, was previously considered to have a wide distribution in the Indo-West Pacific, from the Persian Gulf to southern Japan (e.g. [5]). Although little information exists for this species throughout most of its range, the life history and ecology of this species has been reasonably well studied in Japanese waters, in particular by [6]. *Aetobatus flagellum* became the focus of much attention in the Ariake Bay region of Kyushu Island due to the significant increase in numbers and the destructive effects this species was considered to have on commercial bivalve stocks in this area. Since 2001, ‘predator control’ programs were introduced to reduce the eagle ray population with as many as 10,000 individuals killed annually [6]. In recent years, the populations of eagle rays in Ariake Bay have declined.

While most published information on *A. flagellum* is focused on life history and ecology of Ariake Bay population, little information on its taxonomy was available until now. [7] provided a redescription of *A. flagellum* based on material from the Persian/Arabian Gulf, India, Indonesia and Borneo. These authors stated that, based on molecular and morphological evidence, the northwest Pacific population of *A. flagellum* is likely not conspecific with the true *A. flagellum* and possibly represents a separate undescribed species.

The genus *Aetobatus* Blainville consists of at least four nominal species: *A. flagellum*, *A. laticeps* Gill, *A. narinari* (Euphrasen) and *A. ocellatus* (Kuhl). The latter three species belong to the *A. narinari*-complex of whitespotted eagle rays which require an extensive taxonomic revision to determine how many species are involved. A further seven names are available for members of the *A. narinari*-complex, but no historical names are available for the northwest Pacific species previously considered conspecific with *A. flagellum.* In this study, the northwest Pacific species is formally named and described based predominately on fresh material from Ariake Bay and the Yatsushiro Sea (Kyushu, Japan). Comparisons are made between the new species and its congeners.

## Materials and Methods

### 1. Ethics Statement

Specimens of the new species were obtained from two locations, Ariake Bay and the Yatsushiro Sea. In Ariake Bay, specimens were collected from: commercial gillnet fisheries (Ooura Fishermen’s Cooperative Association, Saga); the predator control program, which also operate gillnets; and from commercial fisheries which operate set nets (Ryuo Fishermen’s Cooperative Association, Saga) and bottom trawls (Shimabara Fishermen’s Cooperative Association, Nagasaki). In the Yatsushiro Sea, specimens were collected from commercial gillnet fisheries (Kagami Fishermen’s Cooperative Association, Kumamoto) and the predator control program. The gill nets used are 10 m high by 400–500 m long and have 20 cm mesh size. They are operated during late spring and autumn, usually in the morning, with operating time dependent on tides. The set nets are small mesh nets which target small shrimps and occasionally have a bycatch of eagle rays. The bottom trawls are permitted to operate from May to August and November to February, targeting shrimps, swimming crabs (Portunidae) and bony fish such as flounder, butterfishes (*Pampus*), and tonguefishes.

No permissions were required to obtain specimens in this study as Ariake Bay and the Yatsushiro Sea are fishing grounds and are not protected. Permission was obtained to use specimens for research by the predator control program’s government and the fisheries cooperative association. We confirm that these activities did not involve endangered or protected species. No approval is required by Nagasaki University for research using moribund bycatch from commercial fisheries or the predator control program. All specimens examined were sacrificed by the commercial fishers or the predator control program operators before being obtained by the authors.

We obtained permission from the National Science Museum in Tokyo (NSMT), Australian National Fish Collection in Hobart (CSIRO) and the Faculty of Fisheries, Nagasaki University (FFNU) to access the collections. One specimen examined at NSMT is a historical specimen in its collection. The other types were collected by two of us (KF, AY) from commercial fisheries and the predator control program and donated to the FFNU (n = 15) and CSIRO (n = 2) collections.

### 2. Specimens Examined

Eighteen specimens of the new species were used for the description of this new species. Most specimens were collected in the field during surveys of Ariake Bay (n = 15) and the Yatsushiro Sea (n = 2), between 2005 and 2013, by two of us (AY, KF). Two of these specimens are deposited at the Australian National Fish Collection in Australia (CSIRO) and the remaining specimens were deposited in the ichthyological collection of the Faculty of Fisheries, Nagasaki University (FFNU). Muscle tissue samples were taken from 5 of these specimens and were stored frozen. Whole retained specimens were injected with concentrated formalin (into gut cavity) and then fixed in a 10% formalin solution. Five additional specimens of the new species, deposited at either the National Science Museum, Tokyo (NSMT, n = 2) or the Hokkaido University Museum of Zoology, Hakodate (HUMZ, n = 3), were also examined.

### 3. Morphology

Full morphometric data was collected for the 18 type specimens of the new species following the methodology proposed by [8] for eagle rays. In addition to the 65 measurements outlined in [8], the following 9 additional measurements were also taken: distance across spiracle openings (taken dorsally, distance between lateral margins of spiracles); lower jaw to anterior cloaca; clasper inner length (from pelvic-fin insertion to clasper tip); clasper outer margin length (from junction of clasper and pelvic inner margin and clasper tip); clasper base width (at base of clasper); distance from edge of disc to 1^st^ gill opening (minimum distance); depth of nasal curtain notch (horizontal distance from posteriormost margin of nasal curtain to centre of notch); width of upper jaw plate (at widest point); width of lower jaw plate (at widest point) (see Table S1). One of the paratype specimens (FFNU-P-2015) was dissected after measurements and images were taken to allow examination of skeletal components, such as the chondrocranium, mature claspers, and pectoral and pelvic girdles.

### 4. Meristics

Counts of radial cartilages and vertebrae were made from dissection (FFNU-P-2015) and from radiographs (NSMT P-65338 and CSIRO H 7465-01). Radiographs of larger type specimens were not possible with the radiograph units available. Four juvenile specimens (<350 mm DW), were radiographed but the vertebrae and fin radials were not adequately calcified to allow counting. The first enlarged anterior element of the pelvic fin (with 2–3 distal segments fused at their bases) is counted as one. Intermediate pectoral-fin radial elements were assigned to a pterygial unit based on the relative level of overlap with each of the adjacent units. The first distal propterygial and metapterygial elements were considered to form part of the main skeleton and were not incorporated into counts. The first synarcual centra are included in vertebral counts. Vertebral counts included in this paper include: synarcual centra, monospondylous centra, pre-dorsal diplospondylous centra and post-dorsal diplospondylous centra. The notochord of the tail was excluded from counts.

### 5. Skeletal Characteristics

One of the adult male paratypes (FFNU-P-2015) was dissected after morphometric measurements were taken. The neurocranium, jaws and tooth plates, scapulocoracoid, pelvic girdle and claspers were removed by dissection and remaining tissue removed using hot water. The dissected skeletal components were preserved directly into 70% ethanol. Skeletal terminology follows [9] and [10]. Outline illustrations were prepared in Adobe Illustrator by tracing images of the skeletal components. It should be noted that the skeletal components of only a single specimen were examined so there is no data on intraspecific variation of these components.

### 6. Nomenclatural Acts

The electronic edition of this article conforms to the requirements of the amended International Code of Zoological Nomenclature, and hence the new names contained herein are available under that Code from the electronic edition of this article. This published work and the nomenclatural acts it contains have been registered in ZooBank, the online registration system for the ICZN. The ZooBank LSIDs (Life Science Identifiers) can be resolved and the associated information viewed through any standard web browser by appending the LSID to the prefix “http://zoobank.org/”. The LSID for this publication is: urn:lsid:zoobank.org:pub: E2AFDF9B-265E-4C9C-9B11-742E8BA3C3B4. The electronic edition of this work was published in a journal with an ISSN, and has been archived and is available from the following digital repositories: PubMed Central, LOCKSS.

## Results

### 1. Diagnosis and Description

***Aetobatus narutobiei*** White, Yamaguchi, and Furumitsu **sp. nov.** urn:lsid:zoobank.org:act: 814AE96E-1405-404E-963E-AE47E3AC3765.

(Figs 1–13; Table S1).

**Figure 1:**
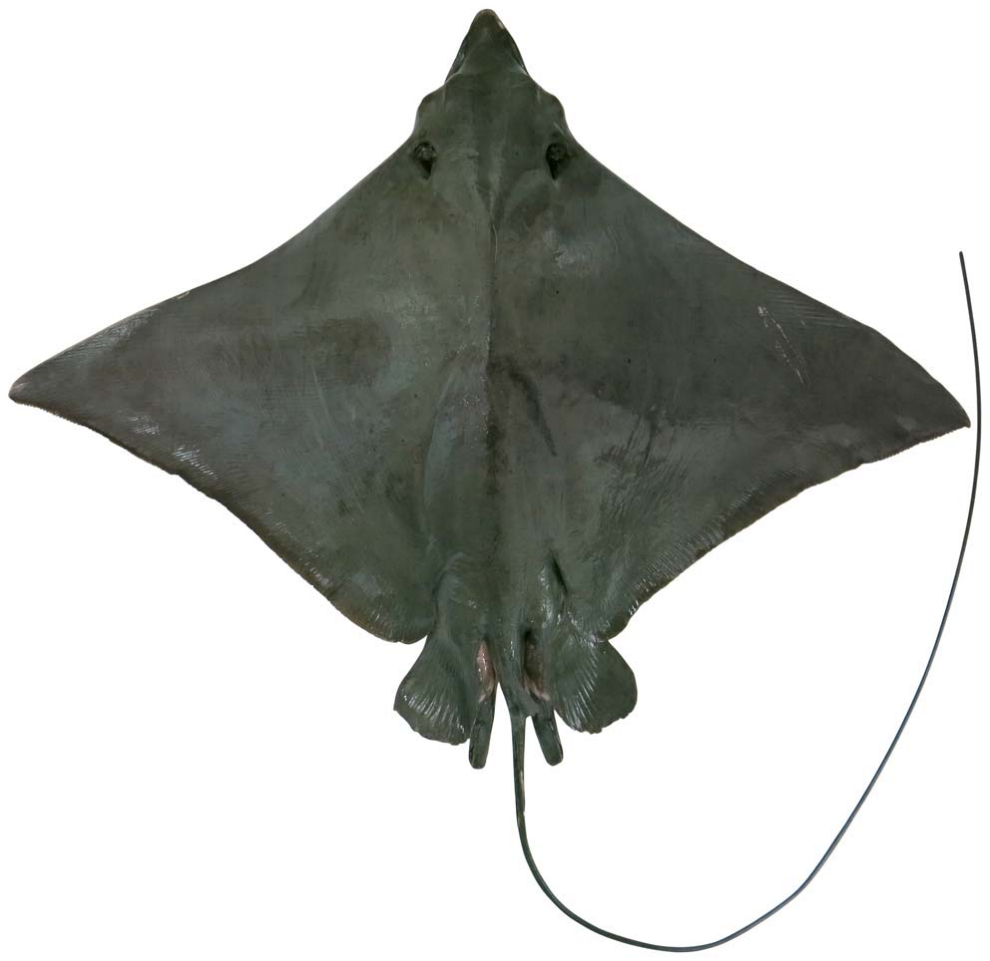
Dorsal view of *Aetobatus narutobiei* (FFNU-P-2001; holotype). Adult male (831 mm DW).

**Figure 2:**
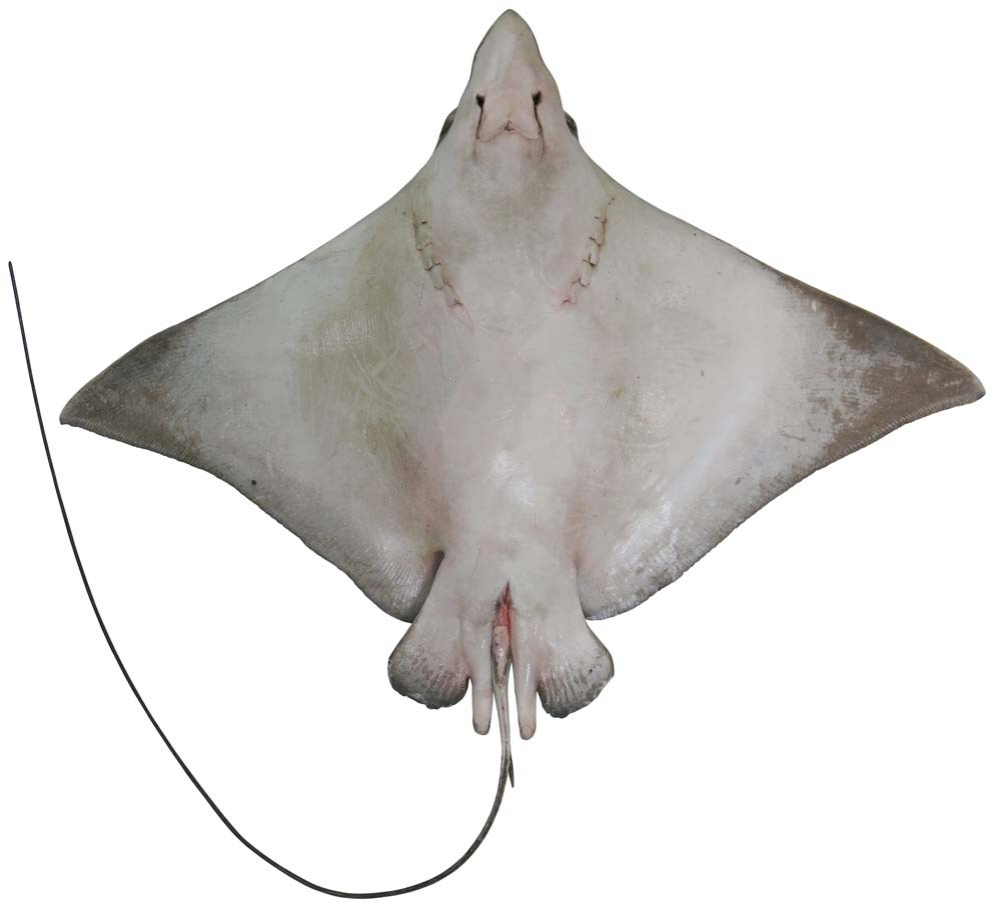
Ventral view of *Aetobatus narutobiei* (FFNU-P-2001; holotype). Adult male (831 mm DW).

**Figure 3:**
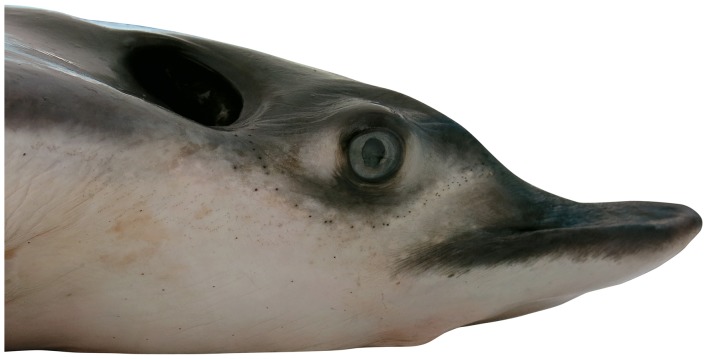
Lateral head view of *Aetobatus narutobiei* (FFNU-P-2001; holotype). Adult male (831 mm DW).

**Figure 4:**
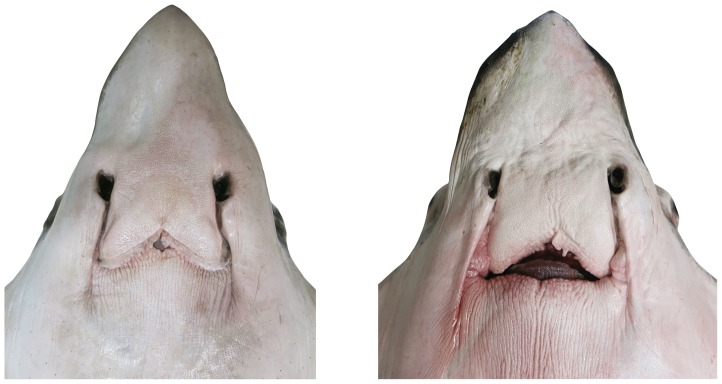
Ventral head view of an adult male and adult female *Aetobatus narutobiei*. A. adult male holotype (FFNU-P-2001; 831 mm DW); B. adult female paratype (FFNU-P-2004; 1210 mm DW).

**Figure 5:**
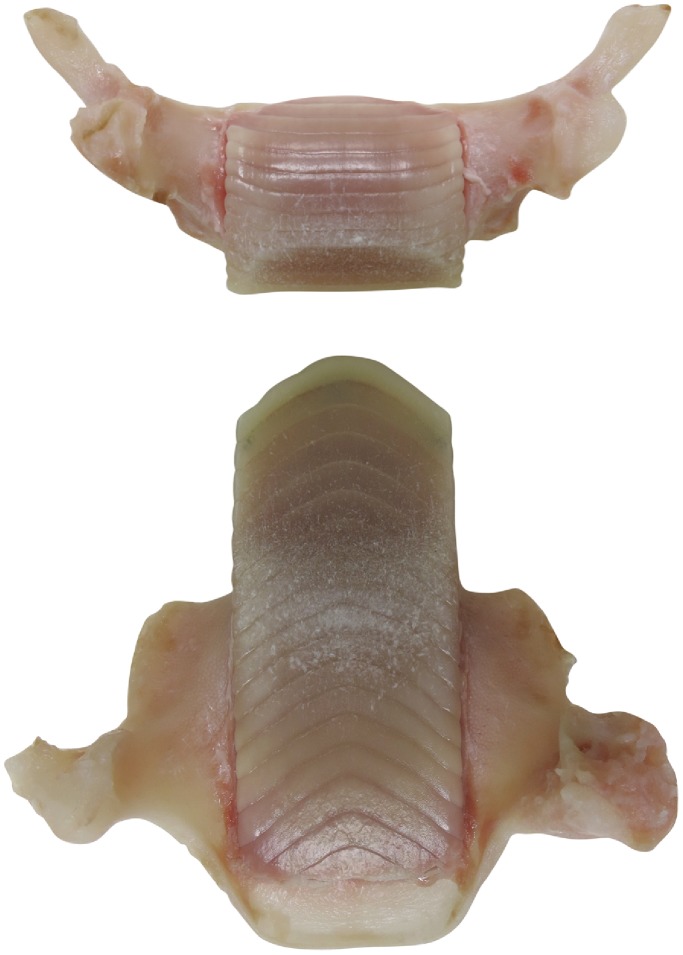
Jaws of *Aetobatus narutobiei* (FFNU-P-2015; paratype). A. upper jaw and B. lower jaw of the dissected adult male (803 mm DW).

**Figure 6:**
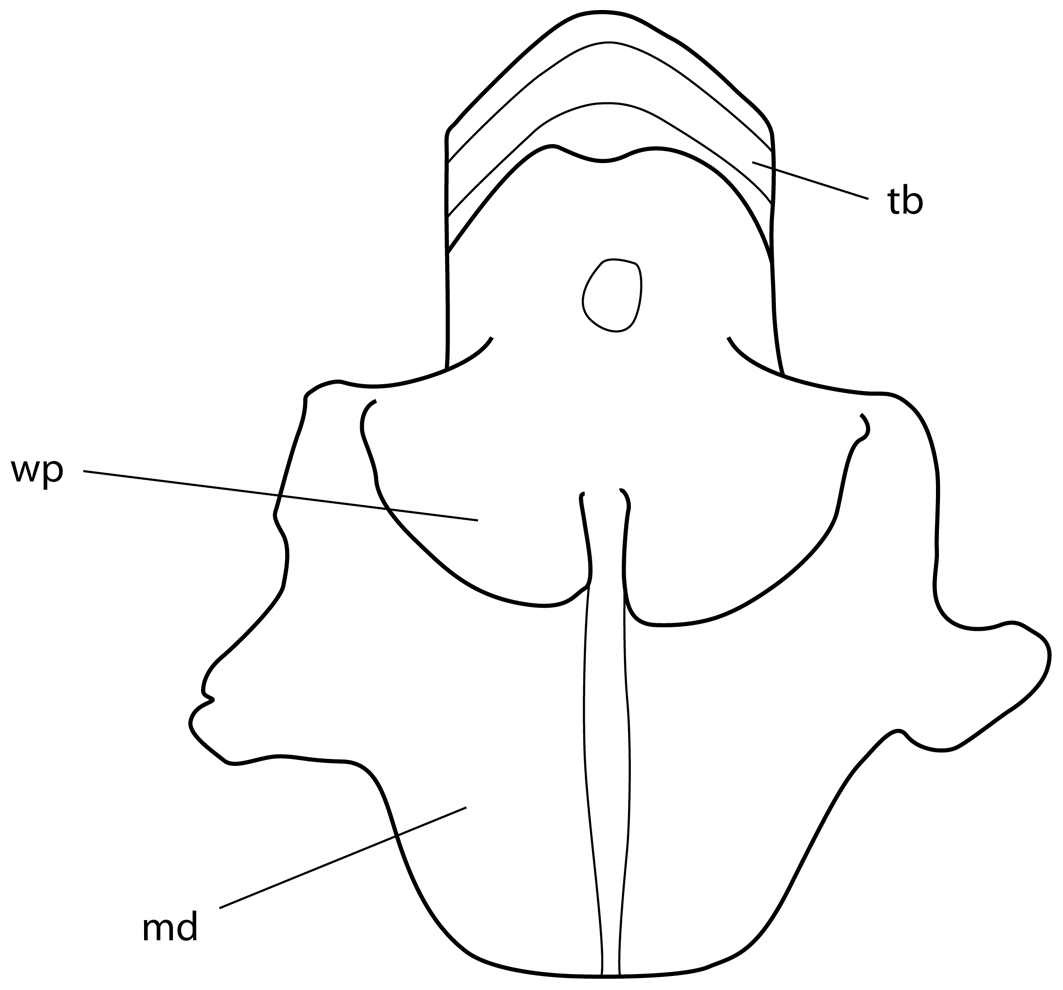
Mandibular arch of *Aetobatus narutobiei* (FFNU-P-2015; paratype). Outline illustration (ventral view). Abbreviations: md – mandibular cartilage; tb – tooth band; wp – wing-like process.

**Figure 7:**
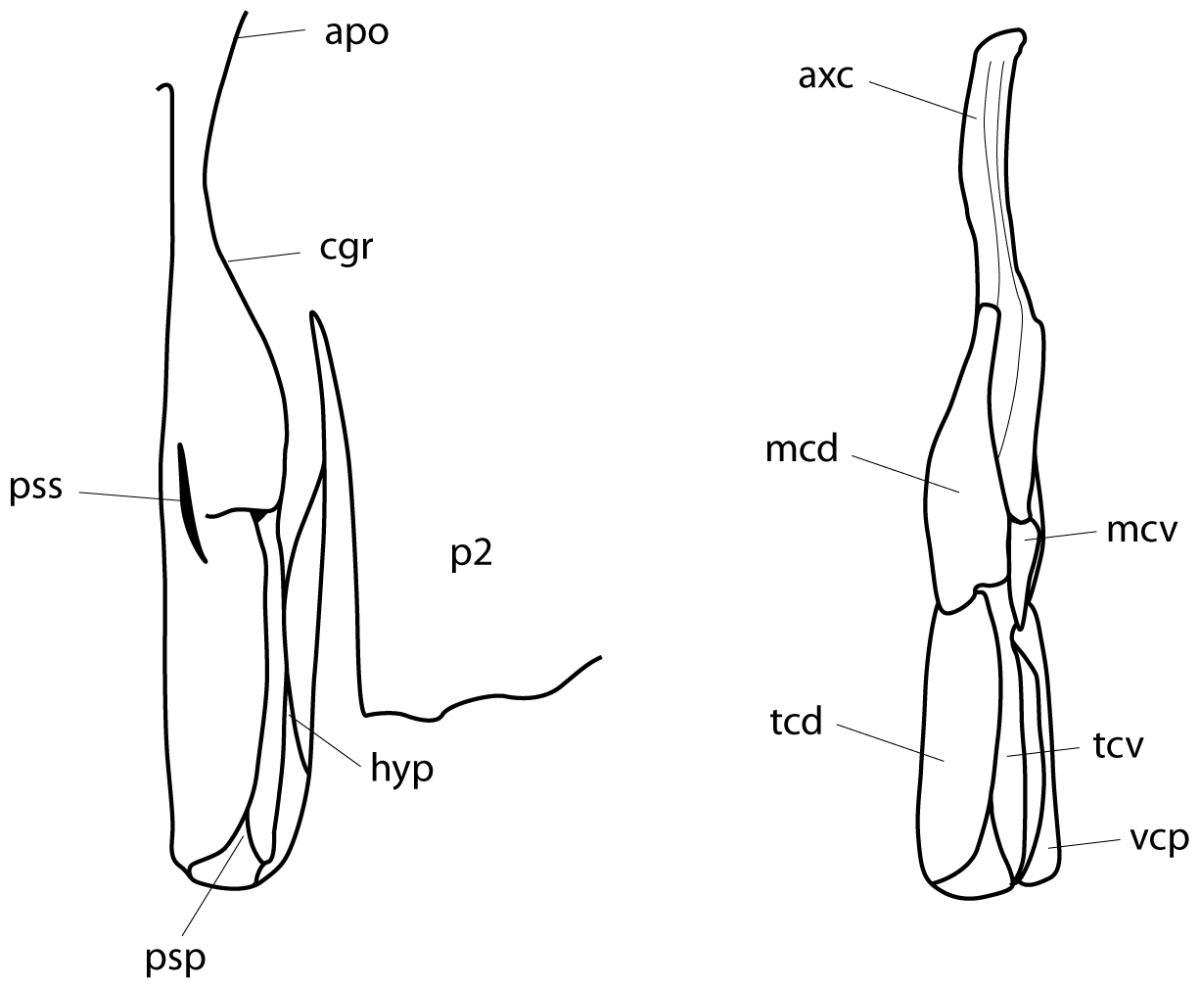
Clasper and clasper skeleton of adult male *Aetobatus narutobiei*. Outline illustration of the left clasper. A: dorsal view of intact clasper (holotype FFNU-P-2001); B: dorsal view of clasper skeleton (paratype FFNU-P-2015). Abbreviations: apo – apopyle; axc – axial cartilage; cgr – clasper groove; hyp – hypopyle; mcd – dorsal marginal cartilage; mcv – ventral marginal cartilage; p2– pelvic fin; psp – pseudopera; pss - pseudosiphon; tcd – dorsal terminal cartilage; tcv – ventral terminal cartilage; vcp – ventral covering piece.

**Figure 8:**
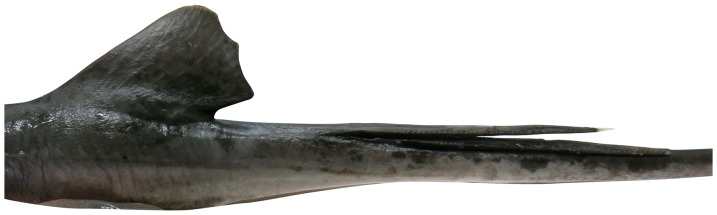
Tail of *Aetobatus narutobiei* (FFNU-P-2001; holotype). Lateral view of the anterior tail of the adult male holotype (831 mm DW).

**Figure 9:**
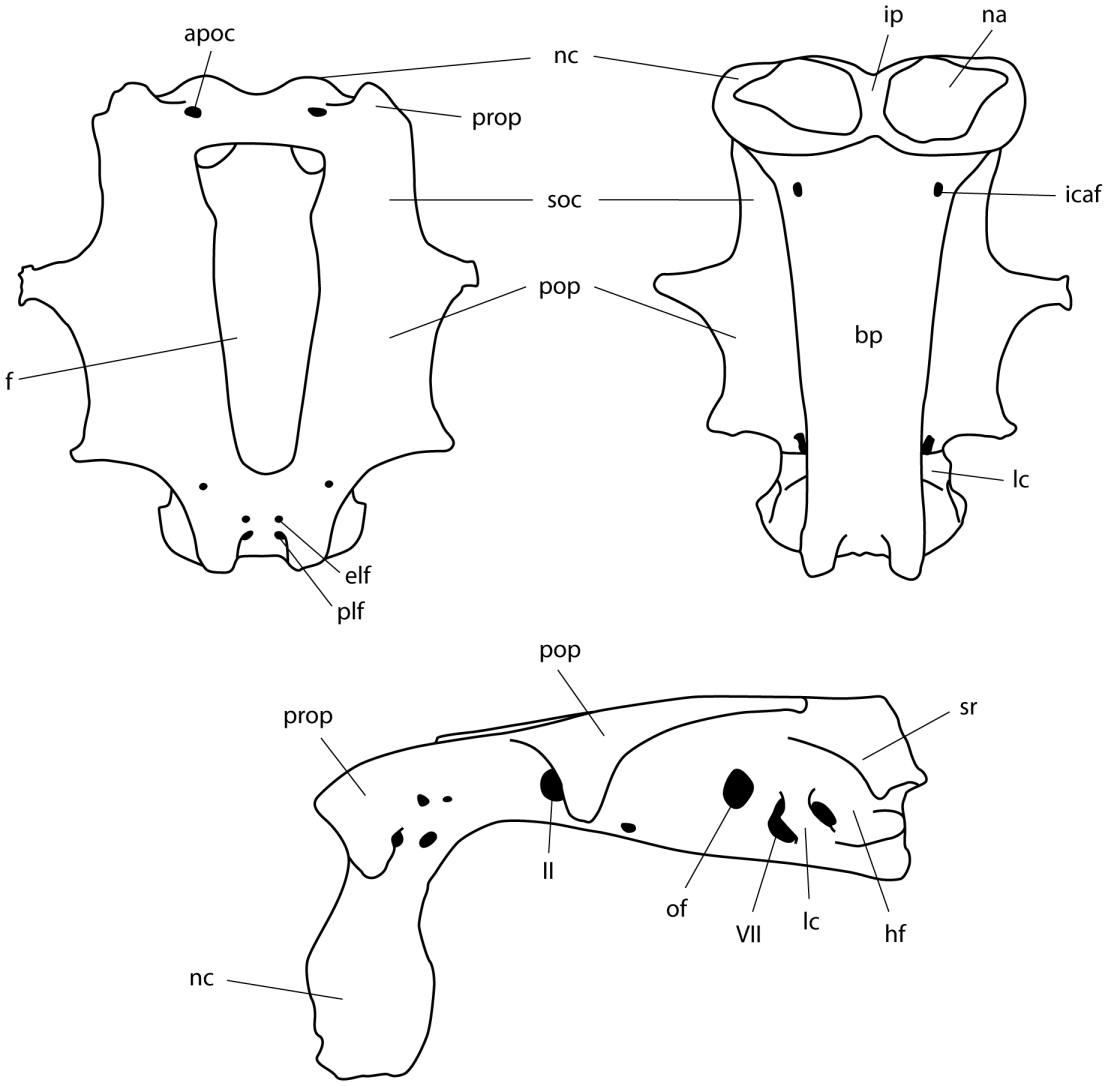
Neurocranium of *Aetobatus narutobiei* (FFNU-P-2015; paratype). Outline illustration. A: dorsal view; B: ventral view; C: lateral view. Abbreviations: apoc – anterior preorbital canal foramen; bp – basal plate; elf – endolymphatic foramen; f – fontanelle; hf – hyomandibular facet; icaf – internal carotid artery foramen; ip – internasal plate; lc – lateral commisure; na – nasal apereture; nc – nasal capsule; of – orbital fissure; plf – perilymphatic foramen; pop – postorbital process; prop – preorbital process; soc – supraorbital crest; sr – sphenopterotic ridge; II – optic nerve foramen; VII – hyomandibular branch of facial nerve foramen.

**Figure 10:**
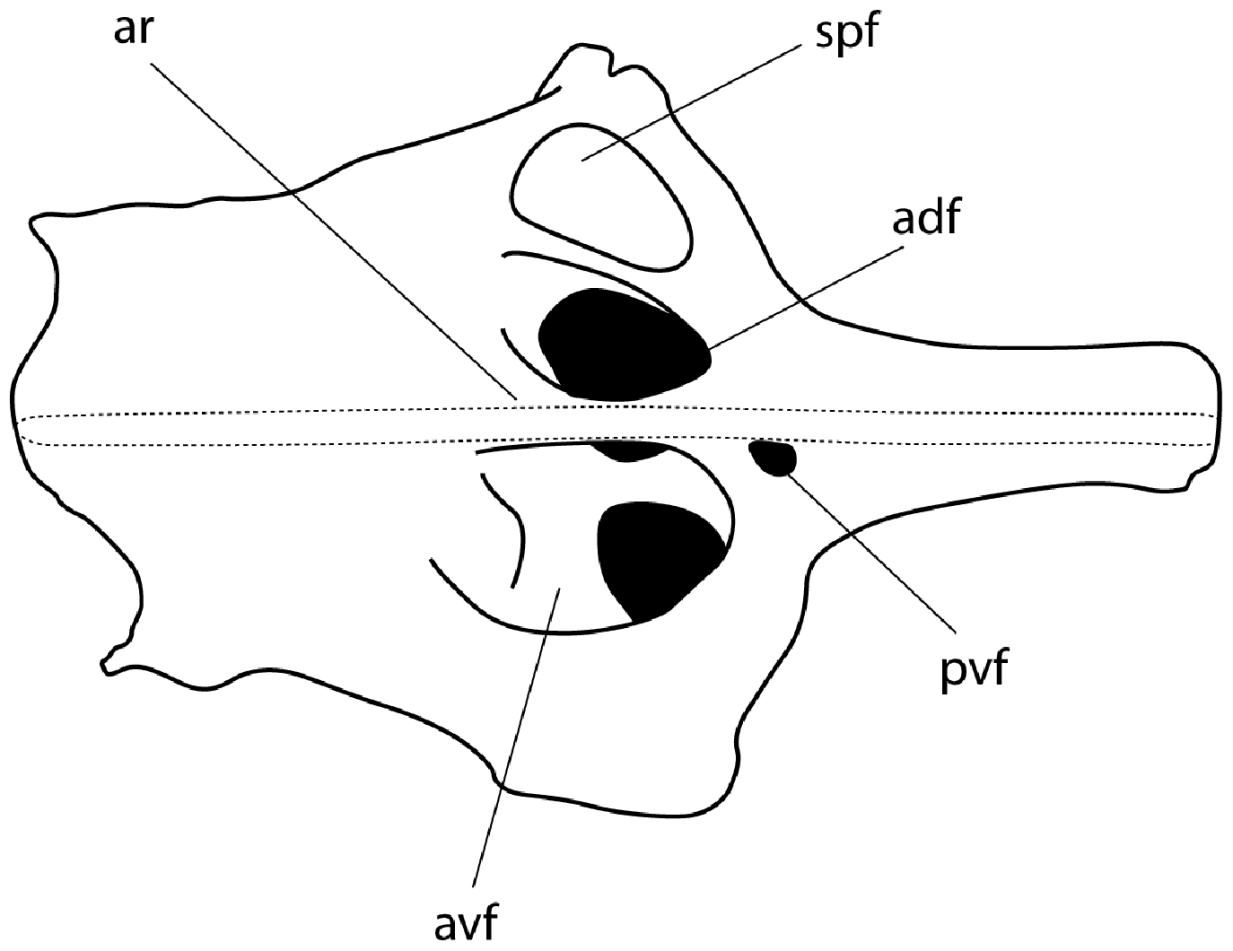
Scapulocoracoid of *Aetobatus narutobiei* (FFNU-P-2015; paratype). Outline illustration (lateral view). Dashed line indicates where pectoral radials are still attached which obscure structures beneath. Abbreviations: adf – anterodorsal fenestra; ar – anterior ridge; avf – anteroventral fenestra; pvf – postventral fenestra; spf – fenestra of the scapular process.

**Figure 11:**
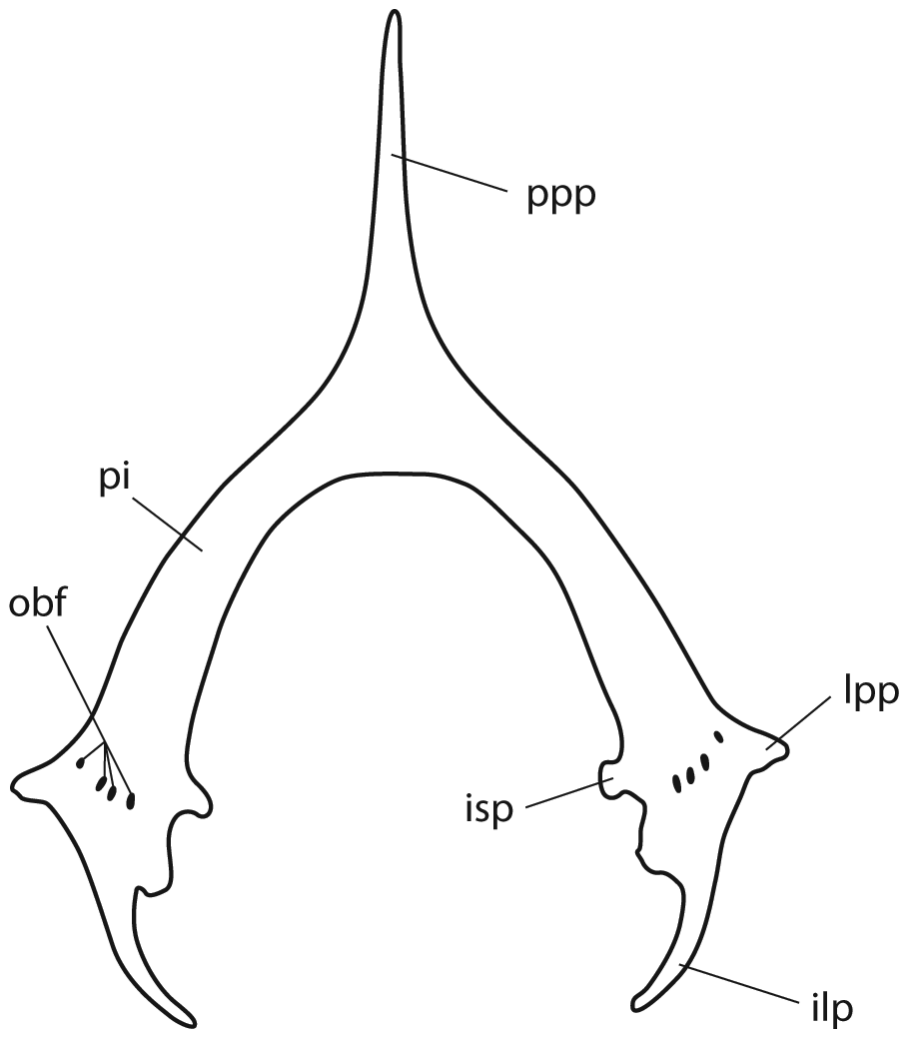
Pelvic girdle of *Aetobatus narutobiei* (FFNU-P-2015; paratype). Outline illustration (dorsal view). Abbreviations: ilp – iliac process; isp – ischial process; lpp – lateral pelvic process; obf – obturator foramina; pi – puboischiadic bar; ppp – prepelvic process.

**Figure 12:**
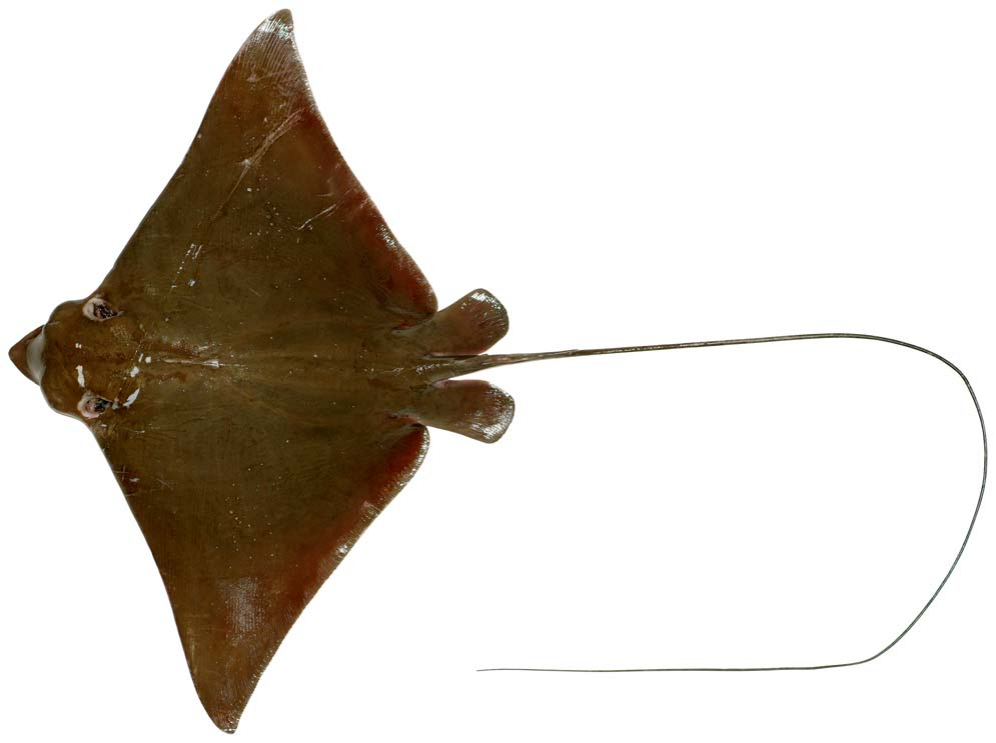
Dorsal view of *Aetobatus narutobiei* (CSIRO H 7465-01; paratype). Juvenile female (516 mm DW).

**Figure 13:**
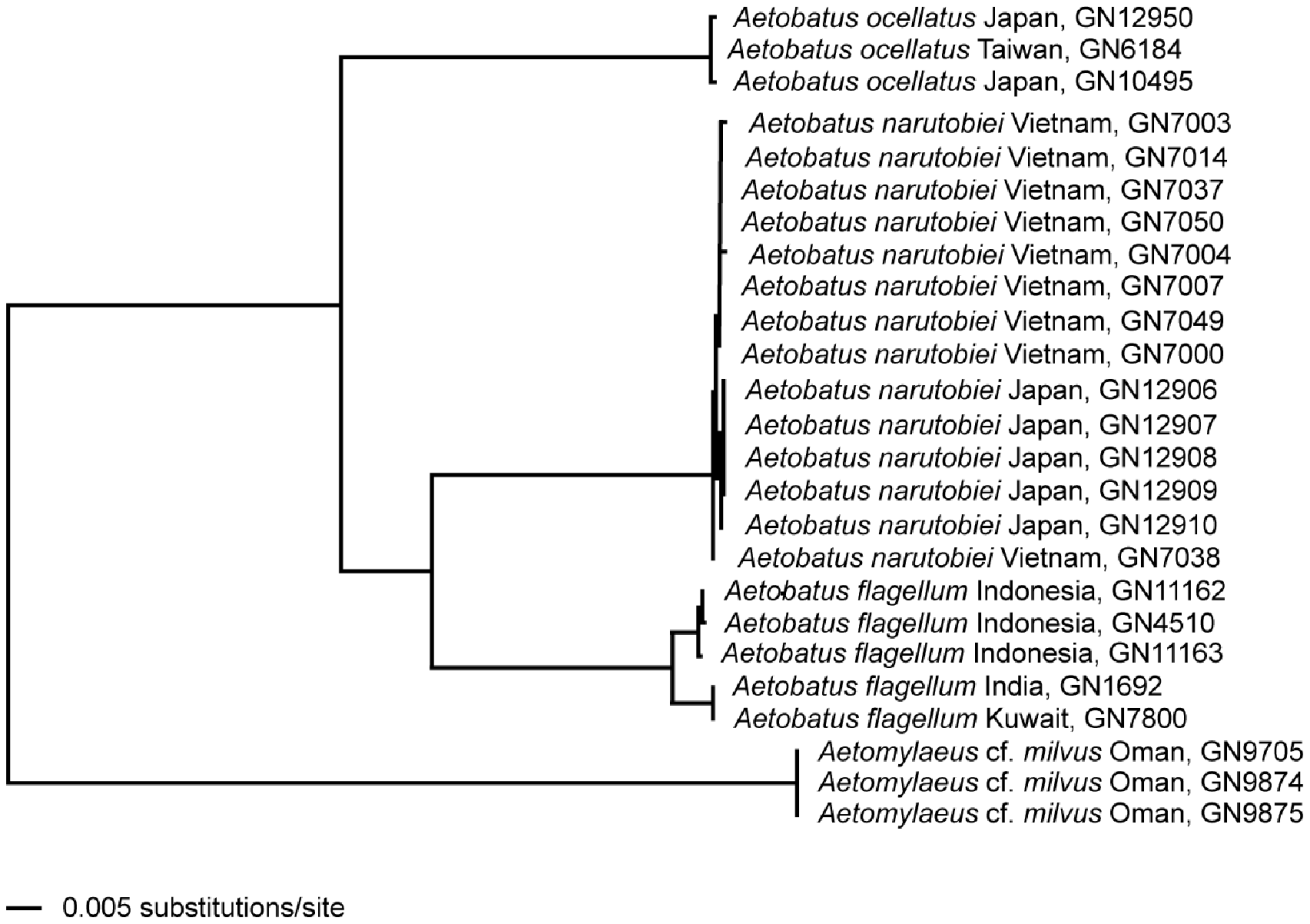
Neighbour-Joining tree for *Aetobatus* species. Based on P distance for Western Pacific *Aetobatus* species, with an *Aetomylaeus* species as an outgroup, using NADH2 sequences.

#### Synonymy

*Aetobates flagellum*: [11]: 198 (“a violet-coloured variety of *Aetobates? flagellum*”) (Macao in July).

*Aetobatis flagellum*: [12]: 82 (China).

*Aetobatus flagellum*: [13]: 1 (Goto Islands, Japan); [14]: 2 (Sea of Japan); [15]: 2 (Seto Inland Sea, Japan); [16]: 143 (Hong Kong); [17]: 143 (Japan); [18], 2001∶450, fig. 239 (China); [6]: fig. 7 (Ariake Bay, Japan); [19]: 53, figs 1–2 (Korea); [20]: 123 (China, South China Sea); [21]: 32 (Okayama Prefecture, Japan); [22]: 28 (Okayama Prefecture, Japan); [23]: 274 (Ariake Bay, Japan); [24]: 1034 (southern Japan); [25]: 89 (Korea); [26]: 10 (Seto Inland Sea, Japan); [27]: 101 (Seto Inland Sea, Japan); [28]: 229 (Japan).

*Aetobatus* sp.: [29]: 85 (Vietnam).

#### Holotype

FFNU-P-2001 (tissue accession GN12906), adult male 831 mm DW, 1577 mm TL, 33°03′05′’ N; 130°12′32′’ E, Ariake Bay, Japan, 5–7 m depth, 3 Aug 2004.

#### Paratypes

CSIRO H 7464-01, neonate male 353 mm DW, 908 mm TL, off Tara, Saga Prefecture, Ariake Bay, Japan, 20 Aug 2010; CSIRO H 7465-01, female 516 mm DW, 1223 mm TL, 33°02′7.2–11.2″ N; 130°19′30.7–14.9″ E, Ariake Bay, Japan, 13.7 m depth, 26 May 2011; FFNU-P-2006, juvenile male 455 mm DW, 1076 mm TL, FFNU-P-2007, neonate male 373 mm DW, 891 mm TL, 33°01′43–47″ N; 130°19′37–45″ E, Ariake Bay, Japan, 7 m depth, 22 May 2006; FFNU-P-2014, neonate female 342 mm DW, 789 mm TL, Ariake Bay, Japan; FFNU-P-2005, neonate male 354 mm DW, 883 mm TL, 32°41′28–43′45′’ N; 130°23′06–24′31′’ E, Ariake Bay, Japan, 58.8 m depth, 16 Nov 2005; FFNU-P-2013, neonate female 352 mm DW, 813 mm TL, off Tara, Saga Prefecture, Japan, 20 Aug 2010; FFNU-P-2010, juvenile female 545 mm DW, 1214 mm TL, FFNU-P-2011, juvenile male 576 mm DW, 1325 mm TL, FFNU-P-2008, juvenile female 556 mm DW, 1220 mm TL, FFNU-P-2009, juvenile female 724 mm DW, 1618 mm TL, FFNU-P-2012, juvenile female 634 mm DW, 1424 mm TL, 33°07′34.9–33.5″ N; 130°19′45.5–58.3″ E, Ariake Bay, Japan, 12.4 m depth, 3 Jun 2010; FFNU-P-2003 (tissue accession GN12908), subadult male 759 mm DW, 1588 mm TL, collected with holotype; FFNU-P-2015 (skeletal components only retained; tissue accession GN12907), adult male 803 mm DW, 1612 mm TL, off Omuta, Fukuoka Prefecture, Ariake Bay, Japan, 13 Oct 2004; FFNU-P-2002 (tissue accession GN12909), adult male 937 mm DW, 1837 mm TL, FFNU-P-2004 (tissue accession GN12910), adult female 1210 mm DW, 2349 mm TL, 32°34′39.15″ N; 130°32′22.93″ E, Yatsushiro Sea, Kumamoto Prefecture, Japan, 6.2 m depth, 29 May 2013; NSMT-P 65338, juvenile female 373 mm TL, 899 mm TL, Totoro fishing port, Nobeoka City, Miyazaki Prefecture, Japan.

#### Other specimens

HUMZ 103757, juvenile male 392 mm DW, HUMZ 103758, female 332 mm DW, HUMZ 103759, juvenile male 386 mm DW, Wakaura Bay, Wakayama, Wakayama Prefecture, Japan, 7 May 1984; NSMT 72366 (deformed specimen), possibly an embryo, female 283 mm DW, off Kanaya, Hamasaka, Hyogo Prefecture, Japan, 21 Oct 2005.

#### Diagnosis

A medium to large-sized *Aetobatus* (attaining 1500 mm DW) with the following combination of characters: dorsal surfaces uniformly greenish grey to brownish, without pale spots or ocelli; head long (ventral head length 27.4–31.9% DW); rostral lobe long to very long (longest in adult males) and narrow, tapering evenly to tip; teeth plates in a single row, those in lower jaw chevron-shaped; width of lower tooth plate about 2.4 times its width and in about 18 series; pectoral-fin radials 100–104 (excluding propterygial radials anterior of eyes); total vertebral centra (including synarcual) 88–90; pelvic-fin radials 1 (with 2–5 fused elements) +17–18; males mature by about 800 mm DW; born at 334–352 mm DW.

#### Description

Disc diamond-shaped (Figs. 1 and 2), broad but relatively short, width about 1.58 (1.54–1.72) times disc length; prepectoral length 3.82 (3.45–4.04) in disc length; axis of greatest width of disc well posterior to scapular region, over abdominal cavity, its horizontal distance from snout tip 1.40 (1.31–1.52) times in distance from tip of snout to pectoral-fin insertion; moderately deep, greatest thickness above scapular region and posterior head, thickness 7.16 (6.51–8.71) in disc width; without denticles, or thorns (several medium-sized specimens, 516 and 634 mm DW, possessed a patch of very low, barely noticeable, fine, widely-spaced denticles on either side of the midline of the posterior disc before the dorsal fin); a short, bony ridge on midline above scapular region. Pectoral fins very large, wing-like, narrowly triangular, weakly falcate; anterior margin concave basally, nearly straight centrally, moderately convex distally; apex narrowly rounded to subangular, pectoral angle 59 (56.2–62.5)°; posterior margin shallowly to moderately concave near apex, almost straight posteriorly; free rear tip very broadly rounded; inner margin convex; length of anterior margin 48.4 (46.5–51.2)% DW, 1.15 (1.07–1.21) times its base length, inner margin 8.17 (5.52–8.31) in its base; origin over anterior quarter of spiracles; apex located at about level with pectoral mid-base; insertion just posterior to or level with pelvic-fin origin, well anterior to dorsal-fin origin; free rear tip partly overlapping pelvic-fin anterior margin.

Head pronounced, moderately deep, short and relatively narrow; projecting well anterior of pectoral-fin origins; subquadrangular in cross-section at pectoral-fin origin; cranial region of head very broadly rounded in dorsoventral view; chondrocranium pronounced above eyes and spiracles; snout abruptly convex anterior of eyes, becoming deeply concave at origin of rostral lobe (Fig. 3); slightly convex ventrally; scattered, widely-spaced minute pores between mouth and about 4^th^ gill slit, becoming more widely spaced posteriorly; direct ventral head length 31.1 (27.4–31.9)% DW, 1.88 (1.37–2.05) times width at pectoral-fin origins, 3.25 (3.39–5.44) times preorbital length (horizontal), 3.53 (2.59–3.50) times interorbital width; preoral snout length 2.13 (1.39–2.13) times mouth width, 2.71 (1.99–2.85) times internarial width, 0.79 (0.56–0.84) times distance between first gill slits; head width at pectoral-fin origin 16.5 (15.5–20.0)% DW, 1.74 (1.57–1.87) times its height. Rostral lobe fleshy, narrow, long (longer in adult males, Fig. 4); narrowly pointed in dorsoventral view with a rounded apex; bluntly pointed in lateral view; covered with widely-spaced, minute pores ventrally; its length 8.48 (4.76–8.13)% DW, 3.67 (3.85–5.84) in head length, its width 1.57 (1.66–2.08) in head width at pectoral-fin origin.

Interorbital space moderately broad, convex but with a broad medial depression, without ridges, denticles or thorns; interorbital width 8.81 (8.91–10.82)% DW, 2.14 (1.64–2.62) times orbit length, 0.63 (0.64–0.74) times head width at mid-eye. Eyes small, subcircular, lateral on head (eyes not visible in dorsal view), angling towards snout tip, diameter 3.15 (2.60–4.20) in spiracle length, 10.14 (8.71–11.99) in head width at pectoral-fin origin. Spiracles large, suboval, situated dorsolaterally on head, just posterior to orbit and above pectoral-fin origin, more visible laterally than dorsally; margins without any protuberances or folds; length 5.14 (4.42–7.79)% DW, 2.35 (2.13–3.06) times width.

Nostril oval with a broad, shallow, fleshy oronasal groove; anterior nasal fold thin, membranous, internal; posterior nasal fold fleshy, extending from central posterior margin to lateral margin of nostril; internarial space 1.92 (1.29–1.92) in prenasal length, 1.74 (1.25–2.08) times nostril length. Nasal curtain large, elongate, lobate, width 1.22 (1.26–1.69) times length; lateral margin weakly concave, smooth edged; posterior margin divided by deep medial notch, bordered by a long, curtain-like fringe, not following contour of lower jaw; posterior margin of each lobe convex with apices rounded; surface covered with many small pores; apex and posterolateral margin recessible within oronasal groove; a pronounced secondary flap present on mid ventrolateral margin, almost rectangular with fringed margin, covers nostril when nasal flap in normal position.

Mouth moderate-sized, transverse, located ventrally, width 6.1 (6.3–8.0)% DW, 0.47 (0.47–0.72) times preoral length, 2.72 (2.34–2.85) in head width at pectoral-fin origin; not strongly protrusible, one or two series of anterior teeth of lower jaw often visible when mouth closed; buccal region intricately papillate; skin on chin and at margin of lower jaw fleshy, strongly furrowed, papillate, indented slightly at symphysis. Teeth in a single row in each jaw, coalesced to form plates (Fig. 5); lower jaw tooth plate length about 2.4 times its width, its width 0.79 (0.70–0.87) mouth width; lower jaw teeth narrow, chevron-shaped, in 18 series (based on dissected specimen FFNU-P-2015); upper tooth plate length about 0.9; upper jaw teeth narrow, mostly transverse but curving posteriorly distally, in 13 series (based on dissected specimen FFNU-P-2015). Roof of mouth with 2 rows of oral papillae; inner row with 9 moderately large papillae in a relatively straight line; outer row with 5 large papillae, middle and outer papillae more anteriorly located than 2^nd^ and 4^th^ papillae (zigzagged), tips of papillae weakly forked. Mandibular cartilage greatly expanded, long, strongly thickened near symphysis; thick, wing-like processes present on ventral surface (Fig. 6).

Gill openings small, elongated S-shaped, forming a weakly fringed lobe laterally; length of first gill slit 1.20 (0.91–1.42) times length of fifth gill slit, 3.13 (3.04–4.55) in mouth width; distance between first gill slits 3.44 (3.05–3.91) times internarial space, 0.53 (0.51–0.63) times ventral head length; distance between fifth gill slits 2.14 (1.87–2.32) times internarial distance, 0.33 (0.32–0.39) times ventral head length.

Pelvic fins moderately large, relatively narrow, subquadrangular, anterior margin slightly concave to almost straight, apex moderately angular, posterior margin moderately convex with scalloped edge, free rear tip rounded angular, inner margin slightly convex; extending well beyond pectoral-fin free tips; pelvic-fin length 17.2 (14.1–18.8)% DW, 1.25 (0.98–1.41) times width across fin bases, inner margin 10.4 (7.7–12.0)% DW. Claspers of adult males relatively short, broad, not tapering distally, apex broadly rounded, outer length 7.2 (6.1–6.9)% DW. Clasper skeleton comprised distally of a shield-like central covering piece which covers the ventral surface of the clasper, and dorsal (plate-like) and ventral terminal cartilages which support the dorsal surface of the clasper; dorsal marginal cartilage well-developed, larger than ventral marginal cartilage (Fig. 7).

Dorsal fin small, strongly raked back (Fig. 8), its origin just posterior to pelvic-fin insertions by about half to two-thirds its fin base; anterior margin almost straight; apex moderately rounded, just posterior to or opposite insertion of fin; posterior margin nearly straight; free rear tip subangular, inner margin short, straight; predorsal length 1.55 (1.49–1.74) in disc width, fin length 6.5 (5.3–6.1)% DW, height 0.50 (0.42–0.56) times its length, inner margin 4.24 (3.78–7.31) in fin length.

Tail very long (shorter in adult specimens), slender, whip-like, its length (from cloaca origin) 1.29 (1.34–1.98) times disc width; tapering gradually at base to stinging spine, and gradually becoming more whip-like beyond sting; base moderately compressed, suboval in cross section at pelvic-fin insertion, tail width at pelvic insertion 0.82 (0.78–0.99) times height; almost quadrangular in cross section near origin of stinging spine, width 0.68 (0.65–0.88) times height at first spine origin; no dorsal skin fold; a weak, low ventral skin fold sometimes present; a weak naked groove on dorsal surface of tail immediately posterior to base of stinging-spine(s), partially housing spines. Stinging spines 1–2, very elongate, slender, moderately broad-based, strongly tapered, mostly serrated laterally except for basal portion (Fig. 8); distance from sting base to pectoral-fin insertion 13.5 (10.9–14.7)% DW; longest stinging spine 9.9 (7.4–13.0)% DW, 1.51 (1.28–2.42) times dorsal-fin length.

Neurocranium flattened, box-like, very rigid, no rostral projection (Fig. 9); nasal capsules thin-walled and forming anterior portion, strongly expanded ventrally; internasal plate narrow; preorbital processes distinct but small; supraorbital crests present, extending from preorbital to postorbital processes; postorbital processes anteriorly located, consisting of an anterior subquadrangular section and an expanded posterior section which are distally fused, anterior section bar-like and protruding ventrally; fontanelle large, continuous, elongate, narrowing slightly posteriorly; two pairs of lymphatic foramina (endolymphatic and perilymphatic foramina) present and posteriorly located; basal plate flat, narrowing posteriorly.

Scapulocoracoid relatively long and high with a narrow posterior extension (Fig. 10); anterodorsal fenestra moderately large with a depression above; anteroventral fenestra large; postventral fenestra small, located close to lateral midline and posterior to anteroventral fenestra; no postdorsal fenestra visible. Pelvic girdle an inverted Y-shape (Fig. 11); puboischiadic bar strongly arched, broadest where obturator foramina are located; prepelvic process very long, very narrowly triangular, almost twice length of puboischiadic bar; iliac processes well developed, relatively long and curving inwards; lateral pelvic processes relatively short but distinct.

Vertebral centra total (including synarcual) 88–90 (n = 3); total (excluding synarcual) 84–86 (n = 3); monospondylous (including synarcual) 35–37 (n = 3); monospondylous (excluding synarcual) 31–32 (n = 3); pre-dorsal diplospondylous 27–30 (n = 3); post-dorsal diplospondylous 25–26 (n = 3). Total pectoral-fin radials (excluding propterygial radials anterior of eyes) 100–104 (n = 2); propterygium (anterior of eyes) ∼10–14 (n = 2), propterygium (posterior of eyes) 11–15 (n = 3), mesopterygium 32–34 (n = 3), metapterygium 55–57 (n = 2). Pelvic-fin radials: 1 (2–5 fused elements) +17–18 (n = 2). Number of spiral valves 30 (based on one dissected specimen, FFNU-P-2015).

#### Colour

When fresh: Dorsal surface of adults uniformly greenish grey (Fig. 1); sometimes with scattered, irregular darkish blotches; dorsal surface of juveniles uniformly brownish (Fig. 12); eye brownish to greenish; dark (dorsal) and pale (ventral) surfaces well demarcated (waterline) at pectoral-fin origin at junction with head; waterline extending anteriorly to mid eye and very slightly onto forehead (Fig. 3); rostral lobe similar to dorsal colouration which extends slightly onto ventral surface (lateral margins dark; Fig. 4). Tail greenish grey to brownish dorsally, paler ventrally with a mottled, diffuse waterline on mid-lateral margin; uniformly dark posteriorly. Ventral surface mostly whitish (Fig. 2); broad brownish margin along most of disc and pelvic fins, junction between brown margin and whitish ventral colour strongly mottled, broadest on posterior margin, narrowest anteriorly; distal third of pelvic fins brownish; rostral lobe mostly whitish (Fig. 4), anteriormost margin narrowly brownish; nasal curtain whitish ventrally, mostly brownish internally except for whitish on the ventral posterior margin.

### 2. Size

Type specimens ranged from 342 to 1210 mm DW. Juvenile males ranged from 353–455 mm DW; subadult male was 759 mm DW; adult males ranged from 803 to 937 mm DW. Females ranged from 342 to 1210 mm DW. [6] reported that females and males of this species (as *A. flagellum*) attain 1500 and 1000 mm DW, respectively, with maximum weights of 50 and 14.4 kg, respectively. They also reported a size at birth of about 352 mm DW with the smallest neonate being 334 mm DW.

### 3. Distribution

The type specimens of *A. narutobiei* were all collected from the main island of Kyushu in Japan. The majority were collected from Ariake Bay, with two specimens collected from the Yatsushiro Sea and a single specimen from off Nobeoka City, Miyazaki Prefecture. Additional specimens from Japan examined include: three specimens from Wakaura Bay, Wakayama Prefecture, near the eastern entry to the Seto Inland Sea, off Honshu; one specimen from off Kanaya, Hamasaka in the Hyogo Prefecture of Honshu in the Sea of Japan. Additional Japanese records of this species have also been recorded from the following locations: Goto Islands, Nagasaki Prefecture off Kyushu [13]; the Seto Inland Sea [15], [22], [26]; Sagami Bay [27]; and Niigata Prefecture, Noto Peninsula, Maizuru Bay, Tachibana Bay, Ise Bay, Kochi [28]. The northernmost record of this species is Akita, Japan Sea but it is very rare in that location [28]. Most records were from the south-western part of Japan. Records of this species from adjacent regions include: Macao, China [11]; Hong Kong [16]; Korea [19], [25]; and Cat Ba Island in Vietnam [29]. The records from Korea and Vietnam are supported by genetic evidence. This species occurs in marine waters from shallow tidal flats to at least 59 m depth. It only occurs in shallow waters when the water temperature is above 15–17°C, and it appears that in winter months they leave the shallower bays for adjacent seas where the water temperature is above 15°C due to the Kuroshio current-derived warm waters [7].

### 4. Etymology

The specific name is in allusion to the common name of this species in Japanese waters ‘Naru tobi-ei’ (pronounced ‘Nar-oo tobee-ay’) where this species is particularly common and the focus of much research. ‘Naru’ is in reference to Naru Island, one of the five major islands in the Goto Islands which are part of Nagasaki Prefecture; where the species was first recorded in Japan by [13]. ‘Tobi-ei’ is the Japanese name used for eagle rays which translates to black kite (a bird). The name is treated as a noun in apposition.

### 5. Vernacular Names

Naru eagle ray (English), 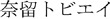 (Japanese), 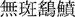 (Mandarin), Cá Ó không châm (Vietnamese).

## Discussion

### 1. Comparisons with Other Species

*Aetobatus narutobiei*, as with its congeners, has the following combination of characters which distinguish it from the other myliobatids in the genera *Aetomylaeus*, *Pteromylaeus* and *Myliobatis*: stinging spine(s) present (absent in *Aetomylaeus*), nasal curtain with a deep central notch (not notched in other genera); pectoral fins joining head above eyes, not continuous with rostral lobe (continuous with rostral lobe in *Myliobatis*; joining head below eyes in *Aetomylaeus and Pteromylaeus*); teeth in both jaws in a single row (7 rows of teeth in other genera). *Aetobatus narutobiei* differs from the white-spotted eagle rays, i.e. *A. laticeps*, *A. narinari* and *A. ocellatus*, in having a uniformly greenish grey to brownish without any white spots or ocelli. The whitespotted eagle ray complex all possess varying patterns of whitish spots or ocelli over most of the disc (sometimes restricted to posterior half).

The ranges for the morphometric characters taken for the new species are quite large, as was also recorded by [18] and [7] for other *Aetobatus* species. Thus, morphometric characters to differentiate species are difficult to find between these species as there is strong overlap between the ranges of the other species, particularly the sister species *A. flagellum*. This highlights the need to examine a good size range and number of individuals when undertaking studies on myliobatid rays, particularly *Aetobatus* species.

*Aetobatus narutobiei* most closely resembles *A. flagellum* but is clearly distinguishable based on the following characters: larger maximum size (∼1500 mm DW in *A. narutobiei* vs. 900 mm DW in *A. flagellum*); larger size at maturity (males become mature between 759 and 800 mm DW vs. between 446 and 543 mm DW); larger size at birth (free swimming at 352 vs. 233 mm DW); rostral lobe of adult males narrowly parabolic and tapering evenly to tip (vs. not tapering posteriorly then abruptly tapering to tip near apex); possibly greater number of tooth series (upper jaw 13 vs. 6; lower jaw 18 vs. 13 [NB based on only a single jaw in each species]); more metapterygial pectoral-fin rays (55–58 vs. 48–54) and total, excluding propterygial rays anterior to eyes, pectoral-fin rays (100–104 vs. 89–96); more pelvic-fin rays (1+17–18 vs. 1+14–16); dorsal fin less raked back with posterior margin almost perpendicular to tail or slanting slightly posteroventrally from apex, its anterior margin length 4.9–5.8% DW (vs. more raked back with posterior margin mostly slanting slightly anteroventrally from apex, anterior margin length 5.6–7.9% DW); slightly greater width across pelvic-fin bases, i.e. 12.1–15.7 vs. 9.7–12.8% DW; lower half of orbit around eye mostly whitish, sometimes greenish to brownish ventrally but whitish between this and the darker upper margin (vs. orbit with brownish margin around entire eye). *Aetobatus narutobiei* also appears to have a much longer lower tooth band than *A. flagellum*, but this is based on only a single specimen of each species and thus intraspecific differences cannot be adequately ascertained.

### 2. Genetic Differentiation

*Aetobatus narutobiei* differs in the sequence of the mitochondrial NADH dehydrogenase subunit 2 genetic marker from other *Aetobatus* species (Fig. 13). The 14 sequences of the new species from Vietnam and Japan grouped closest to, but distinctly different from 5 *A. flagellum* sequences from Indonesia, India and Kuwait. The average pairwise difference between these species was 91–95 nucleotides. The sequences from Japanese *A. narutobiei* specimens had only 1–3 nucleotide differences from the Vietnamese specimens.

*Aetobatus narutobiei* also differs from its congeners in the structure of the mitochondrial cytochrome c oxidase subunit 1 (COI) genetic marker utilised in DNA barcoding based on sequences available in the Barcode of Life Database (www.boldsystems.org). It groups closest to *A. flagellum* but is clearly distinct. Comparison of three COI sequences of *A. narutobiei* from Japan (see [23], M. Sasaki & M. Hamaguchi, unpubl. data) and Korea (see [25]) with 8 sequences of *A. flagellum* from Kuwait and Indonesia (R. Ward, unpubl. data) produced a genetic divergence of between 10.72 and 11.97%. [30] showed that for 210 chondrichthyan species, the average divergence between species within genera was 7.48%, thus this level of divergence between sister species is relatively large.

### 3. Intraspecific Variation

Understanding the level of intraspecific variation within a species is crucial for adequately describing a species. In many cases it is not possible to obtain a good sample size, especially in deeper water species where only low numbers of similar-sized individuals are often available in collections. In this study, a wide size range of specimens was examined and included in the type series in order to help provide a detailed description of this new species. Only a single specimen was available for complete dissection and as a result intraspecific variation in skeletal characteristics could not be investigated.

Comparison of morphometric ranges of *A. narutobiei* highlighted a number of notable differences in morphology between juveniles and subadults/adults. When comparing the morphometric ranges for juveniles (<724 mm DW; n = 13) vs. those for subadults and adults (759–1210 mm DW; n = 5) the following differences were found: longer tail (total length 219.4–257.2% DW in juveniles vs. 189.8–209.2% DW in subadults/adults; tail length 164.5–198.2 vs. 129.1–149.4% DW); shorter head (horizontal head length 26.3–28.9 vs. 30.1–31.3% DW); larger orbit (diameter 4.9–6.3 vs. 3.6–4.4% DW); shorter disc (disc length 58.2–62.6 vs. 62.9–65.0% DW); shorter width across pelvic-fin bases (19.4–24.1 vs. 24.8–26.7% DW); slightly broader head (head width at pectoral-fin origins 16.5–19.7 vs. 15.5–16.5% DW); shorter rostral lobe (length 4.8–6.6 vs. 6.3–8.5% DW).

Comparison between subadult/adult males and the one adult female of *A. narutobiei* also found a number of differences. It should be noted that only a single, large adult female (1210 mm DW) was available to compare to the four subadult/adult males (759–937 mm DW) and more specimens need to be examined in the future to determine whether the following differences are valid. The single adult female differed from the subadult and adult males in the following characters: shorter rostral lobe (6.29 vs. 7.84–8.48% DW; snout to anterior orbit 6.96 vs. 8.04–9.57% DW; prenasal length 7.73 vs. 8.89–9.24% DW); pectoral-fin apices more anteriorly positioned (snout to maximum width 36.94 vs. 41.40–42.34% DW; front of cranium to maximum width 31.40 vs. 32.97–34.78% DW; pectoral-fin posterior margin 50.66 vs. 45.98–49.34% DW); longer pelvic-fin base (base length 10.82 vs. 8.48–9.50% DW); gill slits slightly more widely spaced (distance between first gill slits 17.88 vs. 16.39–16.82% DW).

### 4. Conservation Implications and the Importance of Good Taxonomic Studies

*Aetobatus flagellum* is currently listed as Endangered on the IUCN’s *Red List of Threatened Animals* (www.iucnredlist.org) due to the high level of fishing pressure combined with its inferred naturally low population sizes [31]. This species needs to be reassessed based on new information presented in [7] and the more restricted distribution as a result of the description of *A. narutobiei*. The more restricted distribution of *A. flagellum* and data presented in [7] which highlights that this species prefers coastal areas adjacent to major river flows, means that this species may be of more concern than previously considered. The results of this study and [7] suggest that *A. flagellum* and *A. narutobiei* are not sympatric in any of their range. However, further investigation of the northern limits of *A. flagellum* and southern limits of *A. narutobiei* (e.g. Philippines, Vietnam) are required to accurately determine whether these two species are possibly sympatric in part of their range. The new species, *A. narutobiei*, will also now require an assessment which may be complicated since in part of its range, i.e. Japan, it is considered a pest and is seasonally culled while in other areas there is very little information existing.

This study highlights the crucial role taxonomy plays in the life sciences. Previously, *A. flagellum* was considered a wide-ranging species which although reported to be considered a pest in some areas, is very low in abundance throughout most of the rest of its range. This study, and that of [7], reveals two species are involved, one being undescribed and one being more restricted and likely more threatened than previously considered. New species are always being encountered, especially as fisheries move into deeper waters in recent years. However, what is remarkable in this situation is that more ecological information has been published on *A. narutobiei* than *A. flagellum* yet no taxonomic studies to confirm its identity have been undertaken until now.

## Supporting Information

Table S1 **Morphometric data for** ***Aetobatus narutobiei.*** Morphometric data for the holotype (FFNU-P-2001) with ranges for the 17 paratypes. Measurements expressed as a percentage of disc width.
(XLSX)
